# Case report: coexistence of *C9orf72* expansion and progranulin mutation in a case of genetic frontotemporal dementia—clinical features and neuroimaging correlates

**DOI:** 10.1007/s00415-023-11839-3

**Published:** 2023-06-29

**Authors:** Alma Ghirelli, Edoardo Gioele Spinelli, Elisa Canu, Teuta Domi, Silvia Basaia, Veronica Castelnovo, Laura Pozzi, Giuseppe Magnani, Francesca Caso, Paola Caroppo, Sara Prioni, Cristina Villa, Nilo Riva, Angelo Quattrini, Paola Carrera, Massimo Filippi, Federica Agosta

**Affiliations:** 1 Neuroimaging Research Unit, Division of Neuroscience, IRCCS San Raffaele Scientific Institute, Via Olgettina, 60, 20132 Milan, Italy; 2 Neurology Unit, IRCCS San Raffaele Scientific Institute, Milan, Italy; 3 Vita-Salute San Raffaele University, Milan, Italy; 4 Neurorehabilitation Unit, IRCCS San Raffaele Scientific Institute, Milan, Italy; 5 Experimental Neuropathology Unit, Division of Neuroscience, IRCCS San Raffaele Scientific Institute, Milan, Italy; 6 Fondazione IRCCS Istituto Neurologico Carlo Besta, Unit of Neurology 5—Neuropathology, Milan, Italy; 7 Laboratory of Clinical Molecular Biology, IRCCS San Raffaele Scientific Institute, Milan, Italy; 8 Neurophysiology Service, IRCCS San Raffaele Scientific Institute, Milan, Italy

## Introduction

Frontotemporal lobar degeneration (FTLD) encompasses a group of clinical syndromes characterized by a progressive change in behavior, and/or deterioration of language or executive dysfunction. Presentations include behavioral variant of frontotemporal dementia [1] (bvFTD), primary progressive aphasia [2] (PPA), further classified into semantic (svPPA) and non-fluent variants (nfvPPA). Cognitive symptoms may also be accompanied by motor neuron disease (MND) and/or extrapyramidal signs.

Three different pathologies are associated with FTLD: 90–95% are either FTLD*-*tau or FTLD-TDP, caused by intracellular aggregates of tau or transactive response (TAR) DNA binding protein 43 (TDP-43), respectively [3]. Genetics plays a fundamental role in FTD. Indeed, 20% of cases have a genetic basis and are caused by autosomal dominant mutations; *C9orf72* repeat expansion is the most common genetic cause of FTLD, followed by mutations in the *GRN* and *MAPT* genes [4]. Patients carrying *C9orf72* expansions mostly develop bvFTD and/or MND clinical presentations, accompanied by symmetric and diffuse frontotemporal atrophy, in some cases extending to posterior cerebral regions [5]. *GRN* patients, instead, develop more frequently PPA, accompanied by severe brain atrophy, which is remarkably asymmetric and early involves parietal lobes [5]

A few cases of patients with double FTLD-related mutations have been described, highlighting the necessity for full genetic screening in each individual case [6–8]. Describing cases with two different genetic mutations (pathogenic variants) may help decipher the complex interaction of faulty genes in determining a given disease phenotype. However, none has described the structural brain features of patients carrying double mutations using computational MRI techniques.

In the present study, we describe the case of a patient affected by genetic FTD carrying both *C9orf72* expansion and *GRN* mutation, comparing structural MRI metrics of grey matter (GM) volume with a cohort of eight FTD patients carrying a *C9orf72* expansion, eight patients carrying a *GRN* mutation and 16 healthy controls. We speculate which mutation may be driving the pathogenic mechanism that leads to the symptomatology and the relative specific pattern of atrophy presented by the patient.

## Case description

A 61-year-old man was referred to San Raffaele Hospital in Milan, Italy, with a 1-year history of depressive symptoms which emerged after the patient’s retirement. These were accompanied by reduced initiative, signs of disinhibition, restlessness, and irritability. His relatives sought medical attention after the patient signed up for an online fortune-teller subscription; from that moment they restricted access to his bank account. He also complained of naming difficulties; his spontaneous speech was characterized by frequent anomias, which were overcome with *passepartout* words. He also showed signs of reduced comprehension that worsened with sentences of increasing length. His medical history was positive for hypertension and lumbar sciatica. The patient was right-handed and had 13 years of education. He had a positive family history of cognitive decline (his father, who died at 74). He also had two siblings who were asymptomatic at the time of referral.

On admission to the Neurology Unit, he underwent a full neurologic examination, in which he was alert and oriented in time, space, and person. He recalled 4/5 objects immediately and 3/5 at delayed recall and he remembered major historical events. A mild naming difficulty emerged at spontaneous speech and some apraxia emerged at tasks with his left arm. Frontal release signs resulted positive. He did not show any sign of parkinsonism or MND and the rest of his physical examination was unremarkable. He was still autonomous in basic activities of daily living while needing assistance in some instrumental activities of daily living, such as cooking, shop for groceries, laundry and managing finances.

The patient also underwent a full neuropsychological evaluation (Table 1, for protocol details see Supplementary Information), which described a profile mainly characterized by executive dysfunction, language disturbances, loss of behavioral control, and scarce insight. Specifically, attentional fluctuations and reduced attentional shifting, mental rigidity during problem-solving tasks, and impaired working memory were observed. The low attentional monitoring significantly impacted learning and recall abilities, and the poor verbal working memory markedly affected sentence comprehension and sentence repetition. He presented also with frequent word-finding difficulties during spontaneous speech and naming tests, with a compensative use of *passepartout* words rather than circumlocutions. Concerning the remaining domains, the patient showed preserved visuospatial constructive abilities, semantic knowledge, syntax production during speech and writing, and intact speech, orofacial, and limb praxis abilities.

**Table 1:** Main neuropsychological features of the patients

	Raw score	Adjusted score	Cut-off	Presence of deficit
Global cognition				
MMSE	27	25	24	
ACE-R	67	–	79	+ +
Memory				
Digit span, forward	5	4.92	4.60	
RAVLT, immediate	19	18.6	28.53	+ +
RAVLT, delayed	5	5	4.69	+
Rey’s figure, recall	9	8.25	9.47	+ +
Attention and Executive functions				
Attentive matrices	47	42	31	
Trial Making Test, B-A	166	125	> 186	+
RCPM	29	29.5	18	
Digit span, backward	4	3.87	3.29	
MCST, categories	2	–	3	+ +
MCST, perseverations	21	20.75	> 6.40	+ +
Visuospatial abilities				
Rey’s figure, copy	32	32.5	28.88	
Simple figures, copy	14	14	8	
Clock drawing test	1	–	8	+ +
Language				
CaGi, confrontation naming	39	38.76	41.49	+ +
CaGi, word comprehension	48	48	47.09	
PPT, Object knowledge	48	47.62	40.15	
AAT, repetition	140	–	142	+ +
AAT, reading	29	–	28	
AAT, writing	29	–	27	
Token test, syntax comprehension	25	23.25	26.5	+ +
NAT-I, syntax production	37	–	35.75	
Fluency				
Phonemic fluency	9	6	17	+ +
Semantic fluency	20	20.3	25	+ +
Praxis				
Orofacial praxis	18	–	14	
Ideomotor praxis, right/left	17/19	–	14	
Mood & Behavior				
FBI, Total	11	–	–	+ +
FBI, A	6	–	–	
FBI, B	2	–	–	

Cognitive scores are expressed as raw and adjusted scores (for age, education and sex when possible) according to the normative data (when available). +  + pathological and + borderline performances according to the normative data (see supplementary material for details)

*AAT* Aachener Aphasie Test, *ACE-R* Addenbrooke’s Cognitive Examination-Revised (ACE-R), *CST* Card sorting tests, *FBI* Frontal Behavioral Inventory, *MMSE* Mini-mental state examination, *NAT-I* Northwestern Anagram Test-Italian, *PPT* Pyramids and Palm Trees test, *RAVLT* Rey auditory verbal learning test, *RCPM* Raven’s Coloured progressive matrices

A month later the patient developed symptoms of severe psychosis with delusional thoughts of jealousy against his wife and physical aggressiveness, which prompted admission to the emergency department.

## Diagnostic assessment

**Fig. 1:**
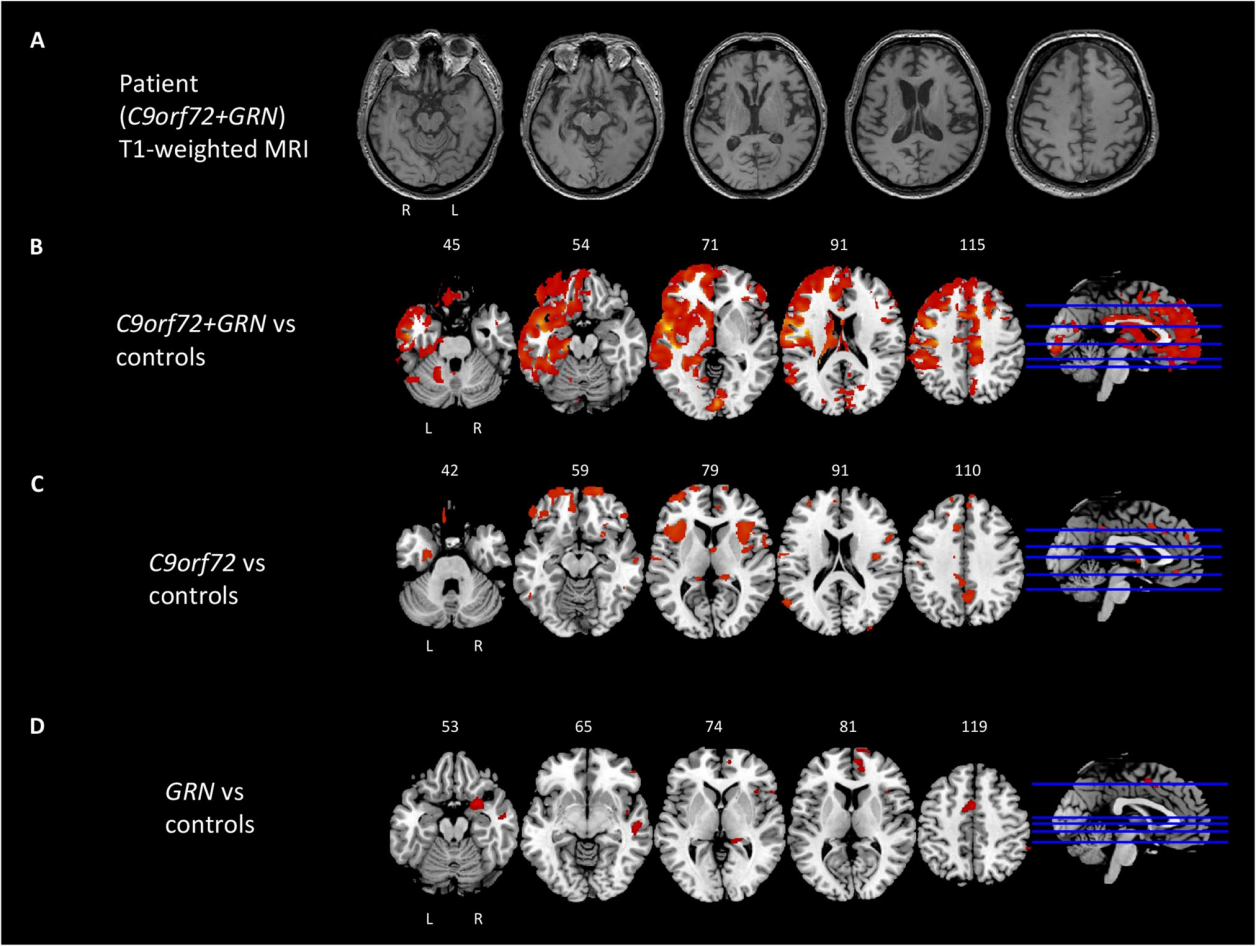
**A** MRI scan showing mild bilateral frontotemporal atrophy with the salience of the left Sylvian cisterns in the case patient. **B** Results of voxel-based morphometry analysis showing regions of significant GM atrophy in proband when compared to healthy controls, **C** *C9orf72* patients compared to healthy controls and **D** *GRN* patients compared to healthy controls. Significant clusters are overlaid on sections of the Montreal Neurologic Institute standard brain. Analyses were corrected for age, sex, and total intracranial volume. Statistical threshold for significance was *p* < 0.05, family-wise error–corrected for multiple comparisons

Visual assessment of the MRI scan showed a mild bilateral frontotemporal atrophy with the salience of the left Sylvian cisterns (Fig. 1a). Lumbar puncture was performed, which presented values in the range of normality (amyloid-beta 42: 925 ng/L [normal values (NV) > 500], total tau: 238 ng/L [0–450], (181) phosphorylated tau; 31 ng/L [NV 0–61], amyloid-beta 42/total tau: 3.88 [NV > 1.27], amyloid-beta 42/(181) phosphorylated tau: 31.8 [NV > 8.1]. A genetic analysis was run and resulted positive for an expansion in the *C9orf72* gene and a missense mutation in the *GRN* gene (p.Cys139Arg) (for details see Supplementary Information). The final diagnosis was of definite bvFTD, based on current clinical criteria and the presence of an FTLD-related genetic mutation [1]. Four years later, his sister developed amyotrophic lateral sclerosis at the age of 56. She was found positive for an expansion in the *C9orf72* gene but did not share her brother’s *GRN* pathological variant. Their other brother instead remained asymptomatic, and no mutation was detected in either of the two genes.

## MRI computational and statistical analysis

For advanced MRI analysis, the case patient was compared with a cohort of eight FTLD patients carrying a *C9orf72* expansion (five with bvFTD, one with FTD/ALS, and two with motor neuron disease) and eight patients carrying a *GRN* mutation (five presenting with bvFTD, and three with nfvPPA). Sixteen healthy controls were also included. The main socio-demographic and clinical characteristics of the subjects are summarized in Table 2**.** Clinical and cognitive data were compared between groups using age- and sex-adjusted analysis of variance (ANOVA) models, followed by post hoc pairwise comparisons, Bonferroni-corrected for multiple comparisons. Analysis was performed with SPSS Statistics 22.0 and the significance threshold was set at p < 0.05. All subjects underwent a 3DT1-weighted scan. Whole-brain voxel-based morphometry analysis was performed to analyze whole-brain grey matter (GM) volume alterations. Volumes of deep GM structures and cerebellar structures were also calculated (for details see Supplementary Information).

**Table 2:** Main socio-demographic and clinical characteristics of subjects

	Patient (C9orf72 + GRN)	C9orf72	GRN	HC	*p*
N	1	8	8	16	–
Age at onset	60	56.9 ± 2.8 [53—61]	60 ± 3.4 [53—65]	–	0.186
Sex (M/F)	1/0	7/1	4/4	7/9	0.161
Scanner (S1/S2)	1/0	4/4	1/7	10/6	0.091
Diagnosis	bvFTD	5 bvFTD, 2 MND, 1 ALS/FTD	5 bvFTD, 3 nfvPPA	–	–
Education (years)	13	10 ± 3.9 [5–13]	8.8 ± 3.4 [5–14]	14.5 ± 2.9 [8—20]	**0.003**
Age at MRI	60	61.2 ± 4.2 [54.9 – 67.1]	62 ± 4.1 [53.7 – 65.2]	59.8 ± 1.7 [57.7 – 63.1]	0.409
Time onset to diagnosis (months)	12	36.1 ± 47.2 [5—119]	29.5 ± 9.2 [23—36]	–	0.873
CDR	0.5	0.8 ± 0.3 [1 – 1]	1.5 ± 1.0 [0.5 – 3]	–	0.441
CDR® + NACC FTLD	–	5.5 ± 4.2 [2.5 – 8.5]	7.8 ± 7.5 [0.5—20]	–	0.714
ALSFSR-r [0–48]	–	36 ± 8.1 [27—43]	–	–	–
MRC sum score	–	88 ± 15.7 [70—99]	–	–	–
Onset bulb/limb	–	1/2	–	–	–
Disease progression rate	–	0.9 ± 0.4 [0.6 – 1.2]	–	–	–

*ALSFRS-r* Amyotrophic lateral sclerosis functional rating scale revised, *CDR* Clinical dementia rating scale, *CDR + NACC FTLD* CDR plus National Alzheimer's Coordinating Center frontotemporal lobar degeneration, *HC* healthy controls, *MRC* Medical Research Council score

**Fig. 2:**
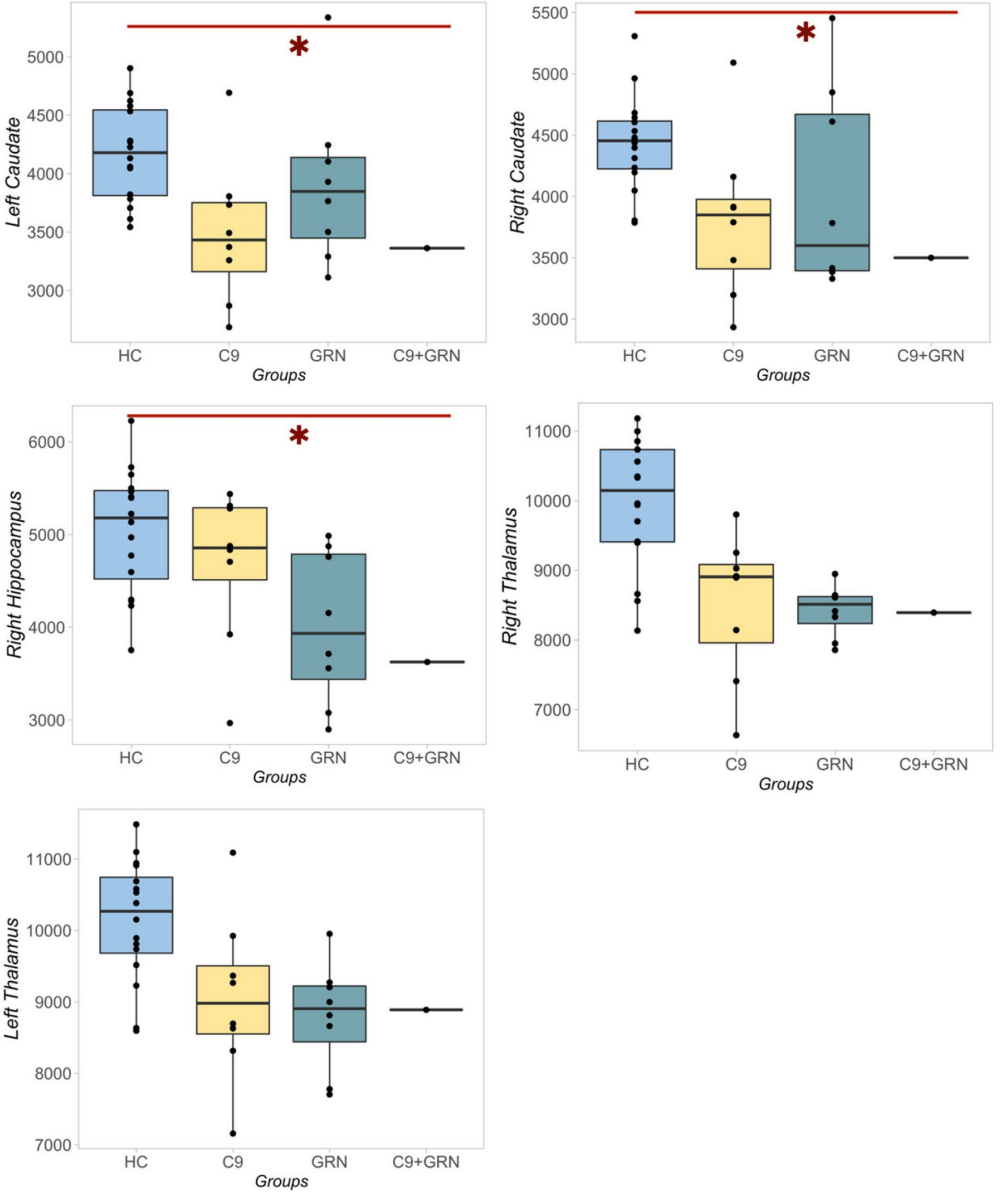
Grey matter volume comparisons between groups, using age-, sex- and MRI scanner-adjusted analysis of variance models, followed by post hoc pairwise comparisons, Bonferroni-corrected for multiple comparisons. *p < 0.05 compared with healthy controls (HC)

At VBM, compared to controls, the proband presented atrophy of the bilateral fronto-temporal cortex, mainly left-lateral, and bilateral involvement of the posterior cerebellum (Fig. 1b). *C9orf72* patients showed bilateral frontotemporal involvement, while *GRN* patients presented a more lateralized left pattern of atrophy (Fig. 1c-d). Compared to controls, the proband showed involvement of left caudate (*p* = 0.037) and right caudate (*p* = 0.017), right hippocampus (*p* = 0.028) and bilateral thalamus (the latter just approaching statistical significance, left *p* = 0.087, right *p* = 0.060) (Fig. 2, Supplementary table). On the other hand, *C9orf72* patients compared to controls presented a similar pattern of atrophy at the level of the left caudate (*p* = 0.021), left (*p* = 0.032) and right (*p* = 0.002) thalamus (Fig. 2, Supplementary table). Lastly, *GRN* patients presented a shared pattern of atrophy compared to controls in the left (*p* = 0.018) and right (*p* = 0.006) dentate, left (*p* = 0.006) and right (*p* = 0.002) thalami and left (*p* = 0.011) and right (*p* = 0.010) hippocampi (Fig. 2**, **Supplementary table). Atrophy at the level of the dentate in *GRN* patients emerged also in comparison with *C9orf72* patients (left dentate *p* = 0.019, right dentate *p* = 0.030).

## Discussion

We reported the case of a patient carrying a double mutation in two FTLD-related genes presenting clinical and neuroimaging features that restate alterations pathognomonic of the two mutations taken separately.

First, the patient presented a complex symptomatology with prevalent behavioral derangements. As it is characteristic of patients carrying *C9orf72* expansions, the case patient developed abnormal behaviors influenced by delusional beliefs [5]; he developed as well depressive symptoms, apathy, restlessness, and disinhibition. Apathy and social withdrawal are the main behavioral symptoms generally developed by *GRN*-mutated patients [5]. The patient presented as well with naming difficulties, distortions, and reduced comprehension. Both *GRN* and *C9orf72* patients can display features of non-fluent aphasia or reduced speech output [5]. However, language impairment is generally more often associated with *GRN* mutations rather than *C9orf72*, and PPA (more specifically, nfvPPA) is more common in individuals carrying a *GRN* mutation as compared to those with sporadic FTD [5].

At VBM, compared to controls, the case patient presented atrophy of the bilateral fronto-temporal cortex, mainly left, and bilateral involvement of the posterior cerebellum. It is known that the pattern of brain damage is remarkably asymmetric in *GRN*-mutated patients [5]. On the other hand, atrophy in *C9orf72*-mutated patients has shown variable patterns, but is usually symmetric and diffuse, with involvement of frontotemporal regions, often including also parietal and occipital lobes [9]. Indeed, in our cohort, *C9orf72* patients showed quite symmetric bilateral frontotemporal involvement, while *GRN* patients presented a more lateralized left-sided pattern of atrophy. Several studies have reported that, compared to non-carriers, *C9orf72* patients exhibit considerable atrophy at the level of the cerebellum [9], which was present also in our proband carrying the two different genetic mutations.

At the level of subcortical nuclei, the index patient showed a pattern of subcortical atrophy of the bilateral caudate, right hippocampus, and bilateral thalamus compared to controls, which was similar to that expressed by *C9orf72* patients (left caudate and bilateral thalamus) and *GRN* patients (bilateral dentate and bilateral thalamus). Other studies have reported the presence of thalamic atrophy as a distinctive sign pointing toward a *C9orf72* expansion. Previous work from our group has demonstrated an inverse correlation between thalamic volumes and behavioral impairments in *C9orf72* patients [10], suggesting that thalamic damage might have a central role in the progression of behavioral impairment in FTD, given the importance of the thalami in cognition and complex behavior [11]. The involvement of the posterior cerebellum, especially in its cognitive/affective regions, is already known to be indicative of the presence of a *C9orf72* expansion. This has been explained as a potential outcome of the cerebello-thalamo-cortical network that connects thalamic structures to the region of the cerebellum involved in the elaboration of emotions and social behaviors [12].

Our report is not the first case of a double FTLD-related mutation reported in the literature. However, this is the first to provide a voxel-based advanced MRI analysis, comparing the proband with groups of patients carrying the single mutations, separately and to rigorously demonstrate volumetric differences between groups. The inclusion of monogenic groups offers cues to speculate on the single contribution of each gene on the complex phenotype of the proband.

A family with two siblings carrying both a *C9orf72* expansion and *GRN* p.Cys31fs frameshift mutation and a diagnosis of FTD-MND was previously described [7]. Post-mortem pathology showed FTLD-TDP type A with TDP-43 positive inclusion and additional p62-positive ‘star-like- inclusions’ in the hippocampus and cerebellum. Another report presented a patient carrying a p.Arg493X mutation in exon 11 of *GRN* and a *C9orf72* expansion who was given a diagnosis of nfvPPA; he had a normal MRI and evidence of reduced cortical metabolism and perfusion in the left frontal lobe and insula at arterial spin labeling MRI [8]. Another patient carrying a *C9orf72* and the p.Tyr294Cys missense *GRN* mutation has been reported, who presented a bvFTD phenotype [6].

Our case patient presented the p.Cys139Arg missense mutation in the *GRN* gene, in addition to a *C9orf72* expansion. Progranulin is a cysteine-rich growth factor that is proteolytically cleaved by elastase to produce seven granulins, which have neuroprotective and neurotrophic roles, as well as anti-inflammatory properties. It is known that plasma progranulin levels predict the pathogenicity of *GRN* variants [13]. Although progranulin plasma levels were not available for our patient, from previous reports it is known that p.Cys139Arg shows progranulin levels below the range detected in healthy individuals and may induce a partial loss of progranulin function [13]. To further prove the pathogenicity of this variant, in vitro functional studies have demonstrated that this mutation causes impaired cleavage of progranulin by elastases. This hampers the production of mature granulins with a consequent reduction in neurite growth-stimulating activity in vitro [14], leading to neurodegeneration. Previous reports of this variant in cases of FTLD confirmed the hypothesis of a possible partial loss of function of the protein and in silico analyses based on evolutionary conservation and protein modeling provided evidence that the p.Cys139Arg variant is most likely pathogenic [15].

Cautiously interpreting data, it is possible that in our case, given that the sister of the patient developed MND carrying only the *C9orf72* expansion, *GRN* mutation acted as a disease modifier, supporting the thesis that pleiotropy plays a major role in the phenotypic determination of FTLD.

These features altogether support the thesis that pleiotropy plays a major role in the phenotypic determination of FTLD.

Study limitations are as follows. First, we included cases acquired with two different MRI scanners, to include the greatest number of mutated patients. However, to overcome this bias, we corrected all analyses for scanner type. Second, *post-mortem* pathological confirmation was not available to better define the neurodegenerative pattern of the patient. Third, genetic analysis for ApoE, PS1, PS2, HMP2B, APP, and PRNP was not performed; also, given the presence of atrophy already at MRI, positron emission tomography was not performed. At CSF analysis, the amyloid-beta 40 test was not tested as it was not implemented yet in our laboratory at the time of the spinal tap. Finally, it was not possible to retrieve information on the rest of the proband’s family pedigree, which would have been interesting in hypothesizing whether one of the mutations was de novo or was inherited.

The description of genetic FTD cases is crucial in disentangling the pattern of disease epicenters and their progression. The present case highlights the importance of providing a comprehensive genetic screening including all FTD genes to patients with a positive family history, considering the possibility of multiple mutations and the consequent prognostic implications for patients and any other proband(s) among family members. Indeed, the advantages in predicting disease trajectories based on the presence of genetic variants are getting clearer as more data are being collected in the literature, as the presence of coexisting mutations could influence disease phenotype. Furthermore, although *post-mortem* pathological confirmation is the gold standard, advanced MRI techniques represent a valuable in vivo instrument to study mutation-specific patterns of degeneration. The rare cases of patients carrying double mutations could help describe the role of each gene and their interactions in undermining different brain functions eventually leading to dementia, expanding the current knowledge on how FTD-related genes disrupt intra- and extra-frontotemporal networks.

### Supplementary Information

Below is the link to the electronic supplementary material.

Supplementary file1 (DOCX 52 KB)

## Data Availability

All data needed to evaluate the conclusions in the paper are present in the paper and/or in the Supplementary Material. Additional data related to this paper may be requested from the corresponding author, upon reasonable request by qualified academic investigators.
