# Supplementary material for: Case report: coexistence of C9orf72 expansion and progranulin mutation in a case of genetic frontotemporal dementia—clinical features and neuroimaging correlates
Source: J Neurol. 2023 Jun 29;270(10):5102–9. doi: 10.1007/s00415-023-11839-3 (PMC10511558; doi:10.1007/s00415-023-11839-3)
Supplement: Supplementary file 1 — Supplementary file1 (DOCX 52 KB) [file 415_2023_11839_MOESM1_ESM.docx]

**Neuropsychological assessment**

A comprehensive neuropsychological assessment was performed by an experienced neuropsychologist and evaluated: global cognitive functioning with the Mini-Mental State Examination [1] and the Addenbrooke’s Cognitive Examination-Revised battery [2]; verbal memory with the digit span forward [3] and the Rey auditory verbal learning test [4]; non-verbal memory with the Rey’s Figure delayed recall [5]; attention and working memory with the attentive matrices [6], the trail making test A and B [7], the Raven’s coloured progressive matrices [8], the digit span backward [3], and the Modified Card Sorting Test [9]; fluency with phonemic [10] and semantic [11] fluency tests; visuospatial abilities with the Rey’s figure copy [5], the simple figures’ copy [6], and the clock drawing test [12]; praxis with orofacial and ideomotor limb apraxia tests [6]; behaviour with the Frontal Behavioural inventory [13].

The patient case also underwent a comprehensive language testing which evaluated: syntactic comprehension with the Token test [14]; syntactic production with the Northwestern Anagram Test-Italian [15]; confrontation naming and single word comprehension with the CaGi test [16]; object knowledge with the Pyramids and Palm Trees Test [17]; repetition, reading, and writing with the Aachener Aphasie Test [18]. For investigating motor speech and speech fluency, we recorded, respectively, multiple repetitions of single words (not standardized) and speech samples while the patient described the image of the picnic picture subtest of the Western Aphasia Battery [19].

**Genetic analysis**

Blood samples were collected from all patients and genomic DNA was obtained and processed in each of the recruiting centers. The presence of GGGGCC hexanucleotide expansion in the first intron of the *C9orf72* gene was assessed using fluorescent amplicon-length analysis and a repeat-primed PCR assay. A cutoff of ≥30 repeats combined with a typical sawtooth pattern was considered pathologic. In addition, *GRN, MAPT, TARDBP, SOD1, FUS, TBK1, TREM2, OPTN*, and *VCP* genes were analyzed by next-generation sequencing and their mutations were confirmed by standard Sanger sequencing. All patients with MND were systematically tested for *C9orf72*, *TARDBP*, and *SOD1* mutations, and additional testing of *FUS* and *TBK1* was performed in the presence of positive family history of MND/dementia. Similarly, all patients with FTD were tested for *C9orf72, TARDBP, MAPT,* and *GRN* mutations, with additional testing of *FUS, TBK1, TREM2, OPTN*, and *VCP* in the presence of positive family history.

**MRI analysis**

All subjects underwent a 3DT1-weighted scan on a Philips 3T scanner (detailed information on the MRI acquisition has been previously published [20]. Whole-brain voxel-based morphometry analysis was performed using SPM12 (fil.ion.ucl.ac.uk/spm/) and diffeomorphic anatomical registration exponentiated lie algebra (DARTEL) registration method [21] to perform a whole-brain analysis of grey matter (GM) volume alterations. Details of the VBM pipeline have been previously described [22]. Gray matter volumes of deep GM structures were obtained using the FIRST tool in FSL (fmrib.ox.ac.uk/fsl/first/index.html), while volumes of the cerebellar vermis and lobules were automatically calculated with atlas propagation and label fusion strategy based on the SUIT atlas [23]. GM volumes were corrected for subject head size via multiplication by the normalization factor derived from SIENAx (fmrib.ox.ac.uk/fsl/sienax/index.html). VBM group comparisons were performed pairwise between groups (proband, C9orf72, GRN and healthy controls) and were tested using ANOVA models in SPM12 adjusting for total intracranial volume, age, sex and MRI scanner type. Results were assessed at p < 0.05, family-wise error–corrected for multiple comparisons. Analysis of subcortical and cerebellar volumes between the proband and other groups was performed using a test for comparing an individual case with a small control sample (i.e, the Crawford-Howell test) [24] using R studio (http://www.rstudio.com/), setting statistical significance at p<0.05.

**References**

1. Folstein, M.F., S.E. Folstein, and P.R. McHugh, *"Mini-mental state". A practical method for grading the cognitive state of patients for the clinician.* J Psychiatr Res, 1975. **12**(3): p. 189-98.

2. Pigliautile, M., et al., *Validation study of the Italian Addenbrooke's Cognitive Examination Revised in a young-old and old-old population.* Dement Geriatr Cogn Disord, 2011. **32**(5): p. 301-7.

3. Monaco, M., et al., *Forward and backward span for verbal and visuo-spatial data: standardization and normative data from an Italian adult population.* Neurol Sci, 2013. **34**(5): p. 749-54.

4. Carlesimo, G.A., C. Caltagirone, and G. Gainotti, *The Mental Deterioration Battery: normative data, diagnostic reliability and qualitative analyses of cognitive impairment. The Group for the Standardization of the Mental Deterioration Battery.* Eur Neurol, 1996. **36**(6): p. 378-84.

5. Caffarra, P., et al., *Rey-Osterrieth complex figure: normative values in an Italian population sample.* Neurol Sci, 2002. **22**(6): p. 443-7.

6. Spinnler, H. and G. Tognoni, *Standardizzazione e taratura italiana di test neuropsicologici.* Ital J Neurol Sci, 1987. **6, suppl 8**: p. 44-46.

7. Giovagnoli, A.R., et al., *Trail making test: normative values from 287 normal adult controls.* Ital J Neurol Sci, 1996. **17**(4): p. 305-9.

8. Basso, A., E. Capitani, and M. Laiacona, *Raven's coloured progressive matrices: normative values on 305 adult normal controls.* Funct Neurol, 1987. **2**(2): p. 189-94.

9. Caffarra, P., et al., *Modified Card Sorting Test: normative data.* J Clin Exp Neuropsychol, 2004. **26**(2): p. 246-50.

10. Novelli, G., Papagno, C., Capitani, E., Laiacona, M., *Three clinical tests for the assessment of verbal long-term memory function: Norms from 320 normal subjects.* Arch Psicol Neurol Psichiatr 1986. **47**(2): p. 278-296.

11. Zarino, B., et al., *A new standardization of semantic verbal fluency test.* Neurol Sci, 2014. **35**(9): p. 1405-11.

12. Manos, P.J., *Ten-point clock test sensitivity for Alzheimer's disease in patients with MMSE scores greater than 23.* Int J Geriatr Psychiatry, 1999. **14**(6): p. 454-8.

13. Alberici, A., et al., *The Frontal Behavioural Inventory (Italian version) differentiates frontotemporal lobar degeneration variants from Alzheimer's disease.* Neurol Sci, 2007. **28**(2): p. 80-6.

14. De Renzi, E. and L.A. Vignolo, *The token test: A sensitive test to detect receptive disturbances in aphasics.* Brain, 1962. **85**: p. 665-78.

15. Canu, E., et al., *Northwestern Anagram Test-Italian (Nat-I) for primary progressive aphasia.* Cortex, 2019. **119**: p. 497-510.

16. Catricala, E., et al., *An Italian battery for the assessment of semantic memory disorders.* Neurol Sci, 2013. **34**(6): p. 985-93.

17. Gamboz, N., et al., *Normative data for the Pyramids and Palm Trees Test in the elderly Italian population.* Neurol Sci, 2009. **30**(6): p. 453-8.

18. Luzzatti, C., et al., *New normative data for the Italian version of the Aachen Aphasia Test (A.A.T.)* Archivio di Psicologia Neurologia e Psichiatria, 1994. **55**(6): p. 1086-1131.

19. Kertesz, A., *Western Aphasia Battery*. 1982, New York: Grune & Stratton.

20. Spinelli, E.G., et al., *Structural MRI Signatures in Genetic Presentations of the Frontotemporal Dementia/Motor Neuron Disease Spectrum.* Neurology, 2021. **97**(16): p. e1594-e1607.

21. Ashburner, J., *A fast diffeomorphic image registration algorithm.* Neuroimage, 2007. **38**(1): p. 95-113.

22. Filippi, M., et al., *Changes in functional and structural brain connectome along the Alzheimer's disease continuum.* Mol Psychiatry, 2020. **25**(1): p. 230-239.

23. Diedrichsen, J., et al., *Imaging the deep cerebellar nuclei: a probabilistic atlas and normalization procedure.* Neuroimage, 2011. **54**(3): p. 1786-94.

24. Crawford, J.R. and P.H. Garthwaite, *Statistical methods for single-case studies in neuropsychology: comparing the slope of a patient's regression line with those of a control sample.* Cortex, 2004. **40**(3): p. 533-48.

**Supplementary table. Volumes of hippocampal, subcortical and cerebellar structures.**

|  | HC | C9orf72 | GRN | Patient (C9orf72 + GRN) | p |
| --- | --- | --- | --- | --- | --- |
| Left Caudate | 4167.07 ± 412.64 (3542.94 – 4902.40 ) | 3489.38 ± 621.23 (2686.82 - 4692.25) | 3910.89 ± 696.42 (31112.09 – 5337.44) | 3362.64 | **0.039** |
| Right Caudate | 4431.60 ± 388.70 (3787.15 – 5307.61) | 3809.79 ± 658.80 (2933.51 – 5091.14) | 4028.22 ± 826.58 (3329.08 – 5454.66) | 3500.16 | 0.070 |
| Left Putamen | 6097.53 ± 623.17 (4696.78 – 7114.56) | 5477.44 ± 1019.55 (4392.05 -7692.93) | 5180.66 ± 827.11 (4104.31- 6321.15) | 5334.04 | 0.056 |
| Right Putamen | 5921.90 ± 607.46 (4791.13 – 6698.13) | 5301.19 ± 1202.21 (3273.58 – 7414.79) | 5230.31 ± 636.18 (4341.53 – 6118.17) | 5088.45 | 0.145 |
| Left Pallidum | 2414.08 ± 415.26 (1627.99 – 3306.63) | 2187.71 ± 504.00 (1509.97 – 2914.52) | 2153.54 ± 329.02 (1619.13 – 2554.77) | 2254.51 | 0.450 |
| Right Pallidum | 2416.99 ± 479.16 (1816.69 – 3383.09) | 2191.26 ± 429.67 (1680.56 – 3092.89) | 2109.89 ± 174.94 (1866.53 – 2385.52) | 2344.21 | 0.339 |
| Left Thalamus | 10137.56 ± 851.26 (8569.19 - 11485) | 9056.02 ± 1164.39 (7157.86 – 11089.68) | 8799.16 ± 757.35 (7705.48 – 9953.70) | 8890.08 | **0.007** |
| Right Thalamus | 9969.52 ± 926.39 (8135.11 - 11181) | 8510.09 ± 1047.02 (6633.16 – 9801.73) | 8421.24 ±.368.24 (7856.41 – 8946.78) | 8392.99 | **<0.001** |
| Left Hippocampus | 5016.27 ± 656.46 (3520.83 – 5958.29) | 4495.26 ± 909.17 (2694.11 – 5652.17) | 3973.59 ± 797.76 (2771.92 – 5008.80) | 4272.97 | **0.027** |
| Right Hippocampus | 5040.11 ± 665.86 (3753.61 – 6227.42) | 4667.63 ± 836.99 (2966.78 – 5437.71) | 4002.89 ± 817.96 (2898.14 – 4986.51) | 3625.89 | **0.016** |
| Brainstem | 28525.11 ± 3281.18 (22242.64 – 34449.98) | 29303.72 ± 3745.57 (22876.41 – 36771.61) | 27178.92 ± 2271.81 (24540.56 – 30953.97) | 26698.17 | 0.559 |
| Left Amygdala | 1912.02 ± 262.11 (1495.42 – 2485.23) | 1804.58 ± 315.01 (1444.82 – 2397.40) | 1664.48 ± 323.56 (1262.76 – 2199.88) | 1886.11 | 0.288 |
| Right Amygdala | 1757.99 ± 281.65 (1251.28 – 2136.46) | 1911.35 ± 272.39 (1369.87 – 2258.45) | 1633.11 ± 188.44 (1377.38 – 1969.14) | 2486.87 | 0.116 |
| Left I-IV | 4167.86 ± 335.85 (3491.20 – 4799.51) | 4425.43 ± 401 (3734.38 – 5160.88) | 4207.26 ± 370.72 (3841.34 – 4804.47) | 4335.47 | 0.425 |
| Right I-IV | 4802.66 ± 399.46 (4050-96 – 5547.69) | 5094.83 ± 405.69 (4571.89 – 5761.01) | 4839.92 ± 447.24 (4456.97 – 5756.75) | 5253.89 | 0.331 |
| Left V | 5421.33 ± 505.39 (4605.02 – 6551.06) | 5879.22 ± 564.89 (4838.24 – 6710.35) | 5451.62 ± 486.34 (4837.93 – 6183.35) | 5581.11 | 0.232 |
| Right V | 3511.28 ± 478.59 (4560.10 – 6359.12) | 5645.77 ± 501.24 (5022.12 – 6414.05) | 5320.16 ± 447.15 (4857.91 – 6129.58) | 5620.82 | 0.390 |
| Left VI | 12031.63 ± 1164.89 (10264.67 – 13968.73) | 12694.25 ± 1304.51 (10980.57 – 14862.84) | 12088.79 ± 1236.65 (10530.52 – 14262.05) | 12282.86 | 0.643 |
| Vermis VI | 2478.87 ± 223.44 (2076.84 – 2764.08) | 2520.83 ± 232.40 (2220.02 – 2831.56) | 2453.48 ± 246.53 (2138.77 – 2848.97) | 2432.45 | 0.940 |
| Right VI | 10485.67 ± 967.83 (9185.53 – 12425.22) | 11105.24 ± 1136.43 (9720.52 -12797.69) | 10361.22 ± 962.44 (9375.93 – 11840.08) | 11276.20 | 0.396 |
| Left Crus I | 17745.67 ± 1417.87 (15410.98 – 20250.55) | 18286.58 ± 2185 (15115.44 – 22365.48) | 17581.91 ± 1553.66 (15693.68 – 19607.66) | 18834.56 | 0.762 |
| Vermis Crus I | 25.94 ± 4.63 (18.20 – 32.45) | 29.72 ± 7.12 (18.98 – 41.28) | 26.85 ± 2.23 (23.95 – 30.09) | 26.47 | 0.385 |
| Right Crus I | 17238.04 ± 1375.14 (14284.25 – 19150.00) | 17334.58 ± 2045.67 (14122.35 – 20572.45) | 16552.05 ± 1994.00 (13659.57 – 19668.98) | 18956.66 | 0.520 |
| Left Crus II | 13000.75 ± 1226.97 (10940.78 – 15520.50) | 13516.33 ± 1699.33 (11647.93 – 17237.93) | 12880.63 ± 1487.65 (10549.02 – 14908.77) | 12326.23 | 0.742 |
| Vermis Crus II | 530.61 ± 51.07 (402.88 – 614.77) | 537.99 ± 89.38 (376.91 – 645.24) | 525.38 ± 60.19 (435.82 – 609.04) | 595.62 | 0.669 |
| Right Crus II | 12271.31 ± 1144.88 (10445.62 – 14499.72) | 12303.09 ± 1505.80 (10869.51 – 15349.66) | 11632.48 ± 1292.68 (9706.52 – 13495.68) | 11886.52 | 0.665 |
| Left VIIb | 6569.33 ± 623.68 (5385.66 – 7851.26) | 6773.22 ± 984.73 (5517.94 – 8710.33) | 6471.30 ± 984.52 (5517.40 – 8710.47) | 6333.19 | 0.865 |
| Vermis VIIb | 237.58 ± 35.69 (169.22 – 305.38) | 239.29 ± 49.10 (171.64 – 321.48) | 227.94 ± 29.39 (181.51 – 273.49) | 258.83 | 0.840 |
| Right VIIb | 6688.95 ± 595.24 (5651.29 – 7752.44) | 6677.83 ± 908.24 (5383.27 – 8459.73) | 6322.45 ± 687.34 (5352.44 – 7439.98) | 6493.79 | 0.663 |
| Left VIIIa | 6941.32 ± 595.72 (5829.44 – 8186.34) | 7054.83 ± 954.42 (5791.13 – 857.33) | 6860.84 ± 779.03 (5607.61 – 7826.57) | 6789.01 | 0.955 |
| Vermis VIIIa | 1512.83 ± 112.23 (1322.56 – 1759.03) | 1555.70 ± 222.61 (1295.64 – 1957.27) | 1478.63 ± 228.77 (1228.34 – 1783.81) | 1460.35 | 0.842 |
| Right VIIIa | 6356.19 ± 534.80 (5393.67 – 7178.04) | 6428.39 ± 851.39 (5247.28 – 7939.44) | 6206.73 ± 781.36 (5259.23 – 7612.64) | 6228.93 | 0.925 |
| Left VIIIb | 5624.79 ± 444.74 (4886.68 – 6337.18) | 5860.47 ± 648.48 (5152.61 – 7263.45) | 5453.72 ± 637.12 (4691.72 – 6405.55) | 5523.02 | 0.592 |
| Vermis VIIIb | 772.97 ± 56.08 (655.67 – 880.84) | 784.18 ± 136.92 (562.38 – 950.38) | 749.38 ± 113.33 (616.57 – 929.78) | 780.18 | 0.903 |
| Right VIIIb | 5373.01 ± 407.78 (4714.12 – 6132.71) | 5482.55 ± 511.92 (4762.49 – 6498.71) | 5140.91 ± 655.66 (4434.59 – 6271.03) | 5278.16 | 0.583 |
| Left IX | 4323.75 ± 492.48 (3170.71 – 5048.91) | 4534.39 ± 577.64 (3768.34 – 5390.95) | 4138.39 ± 586.46 (3561.14 – 5062.84) | 4175.17 | 0.530 |
| Vermis IX | 897.77 ± 118.62 (711.64 – 1160.63) | 918.28 ± 146.56 (737.62 – 1138.29) | 889.50 ± 122.65 (762.15 – 1078.92) | 862.53 | 0.957 |
| Right IX | 4489.09 ± 475.28 (3518.29 – 5283.41) | 4739.83 ± 502.08 (4238.24 – 5591.34) | 4360.97 ± 656.05 (3637.77 – 5292.45) | 4574.45 | 0.547 |
| Left X | 894.55 ± 85.06 (778.55 – 1049.71) | 938.64 ± 115.81 (781.32 – 1179.34) | 930.81 ± 111.35 (788.03 – 1097.32) | 952.98 | 0.694 |
| Vermis X | 484.57 ± 84.76 (364.62 – 655.73) | 520.72 ± 75.43 (463.12 – 657.99) | 533.11 ± 104.84 (409.99 – 698.13) | 492.33 | 0.578 |
| Right X | 884.35 ± 98.34 (749.24 – 1140.14) | 940.73 ± 97.54 (813.24 – 1114.15) | 923.56 ± 98.46 (795.51 – 1086.30) | 1007.39 | 0.403 |
| Left Dentate | 2193.47 ± 249.25 (1906.24 – 2663.35) | 2231.81 ± 141.97 (1066.73 – 2419.66) | 1930.52 ± 140.45 (1737.24 -2167.21) | 1886.11 | **0.014** |
| Right Dentate | 2504.33 ± 329.24 (1963.65 – 3063.95) | 2482.14 ± 184.39 (2224.29 – 2780.09) | 2115.32 ± 161.41 (1934.13 – 2374.02) | 2358.17 | **0.015** |
| Left Interposed | 306.73 ± 51.77 (218.42 – 398.80) | 293.46 ± 22.4 (260.25 – 325.34) | 270.90 ± 38.08 (217.24 – 350.66) | 263.25 | 0.263 |
| Right Interposed | 342.56 ± 63.44 (247.02 – 465.34) | 335.24 ± 27.91 (291.78 – 371.13) | 288.40 ± 28.88 (241.83 – 326.88) | 332.367 | 0.109 |
| Left fastigial | 58.88 ± 10.85 (48.68 – 80.10) | 61.52 ± 10.85 (42.32 – 77.83) | 50.51 ± 10.00 (43.32 – 77.83) | 49.26 | 0.131 |
| Right fastigial | 61.78 ± 13.66 (39.79 – 92.72) | 61.73 ± 10.24 (50.18 – 77.83) | 53.75 ± 8.82 (39.20 – 66.81) | 61.76 | 0.442 |

Values (mm^3^) are reported as means ± standard deviations [min. value – max. value]. P values refer to age-, sex- and MR scanner-adjusted ANOVA model, followed by post-hoc pairwise comparisons, Bonferroni-corrected for multiple comparisons. Abbreviations. HC=healthy controls.
